# Diabetes Mellitus

**Published:** 1909-02

**Authors:** J. C. Densten

**Affiliations:** Scranton, Pa.


For Texas Medical Journal.

### Diabetes Mellitus.

BY J. C. DENSTEN, M. D., SCRANTON, PA.

### ETIOLOGY.

The etiology of diabetes mellitus often lies in obscurity in so much as proving the rationale of the phenomena, yet we venture a few suggestions on the etiology, diagnosis and physiological therapy of this disease, and offer from our own personal observation and experience a few useful and practical hints.

### SYMPTOMS.

Every practicing physician, whether in city or country, has under his observation some time in his practice one or more individual sufferers from diabetes mellitus, and since the symptoms are not only slow and insidious in their outset, but vague and varying in their phenomena, it becomes difficult to diagnose the disease in its incipiency, without resorting to urinalysis, which act the physician generally omits until later.

Now this analysis of the urine is the only key which will unlock the mysterious vault of glucohemia, and by the presence of an excessive amount of sugar in the urine, prove that the term is not a misnomer, and confirms our diagnosis.

### DIAGNOSIS.

When once our diagnosis is confirmed and established, what should be our procedure toward effecting in our patient a return to normal health? We must aim to correct the metabolism. How was this change from the normal brought about, and why continued? We find this condition prevalent alike among the rich and poor, the plethoric and the anemic, while the phenomena in each point to the same metabolism; the same chemical metamorphosis, each case being aggravated by nervousness, worry, great joy or great sorrow. Does this fact not give us some hint of the etiology of diabetes mellitus?

We know that the carbohydrates are not oxidized in whole or in part within the animal economy, but why they are not should be the sceret mission of every physician to investigate and learn.

Does the nervous fluid to and from the brain bear any importance to the subject? I ask this question because we find that many epileptics, after a convulsion, pass an excessive amount of sugar in their urine, and particularly those whose diagnosis points to a tumor somewhere along the spinal tract, or base of the brain, obstructing the normal flow of the nervous fluid to and from that organ. This fact is also to be regarded, viz., that many epileptic seizures are brought about by some metabolic nerve condition, joy, sorrow, fear or excitement, alike often precipitating an attack.

We also find that many of our diabetic subjects are from the ranks of the business and financial world of nervous worries, fast easters and vitiated air breathers.

The etiology of this disease, although seemingly obscure, may often be discovered by an intelligent plunge into the previous life, habits, customs, and acquired so well as the history of hereditary diseases. An existing syphilis may be producing a gumma along the floor of the fourth ventricle, or we may have a gouty or rheumatic diatheses, or find an hypertrophied pericardium, or an endocarditis, or the etiology may lie in faulty metabolism of some organ or viscus as the heart, aorta or pancreas, or there may be cirrhosis of the liver from alcoholic, malarial, or other infection, from which metabolic condition there may appear subjective symptoms, as anasarca, jaundice, ascites, nervous cough, aneurism, epileptic convulsions, etc. When we suspect the etiology of diabetes mellitus to lie in any of these phenomena, we should direct our treatment toward the removal of the cause.

### TREATMENT.

In the treatment of diabetes mellitus, the consensus of opinion seems to have been to pay more attention to hygiene, regime and regimen, than to drug therapy; this, perhaps, because drugs administered seemed to do little or no good, while regimen, by cutting out, in whole or in part, the carbohydrates from the diet and administering the proteins, lessened the sugar output and hence gave better outward results; at least, in the sugar test. There are still others who combine drug therapy and regimen with most satisfactory results.

REGIMEN.

If food be limited to proteins, we are adding tissue-building material, with little or no carbon to supply needed normal body-heat, and energy; hence we are not benefiting the patient—objectively. We are only lessening the amount of sugar-producing material in the system, relieving the kidneys from working overtime perhaps, but requiring more $H_2O$ to supply this needed heat, which in turn combines with the fats already stored in the body and the combustion resulting will of necessity lessen the avoirdupois of the patient; hence we expect, under such procedure, a loss in weight as soon as the carbohydrates are withdrawn from the food. The patient is feeding on himself.

The treatment which is to cure our diabetics should be administered, not with the view of lessening the sugar in the urine by the withdrawl of the carbohydrates from the diet and hence by starvation, but to altering the metabolic condition at the seat of the disease, and restoring to a normal condition those deranged functions which fail in their mission to act as in previously normal health.

DRUG THERAPY.

Arsenic has a beneficial effect in causing a diminution in the sugar output in some cases, as has opium and its alkaloids, morphia and codeine. These are sometimes prescribed alternately —codeine in ¼-grain doses and arsenic in form of Fowler's solution.

The scientific physiological therapy of the action of these drugs lie *in vacuo,* and the knowledge gained by their administration remains a posteriori or empirical; but that they do act beneficially in some cases is certain, and where there administration has been combined with daily baths and proper regimen, have been known to produce in the urine a return to normal, which has continued for months, but which disease, sooner or later, has returned even when the treatment was continued during the interval.

In any treatment for diabetes mellitus, the emunctories must not be neglected—frequent baths are highly necessary, and the bowels and kidneys should be frequently flushed. The nervous system should be tranquilized, and, when possible, a change of business and associations, with plenty of fresh, pure water and air, with deep and prolonged inhalations.

In all cases of this disease there is to be found in the urine an excessive amount of phosphates, which fact evidences great nervous friction, and demands additional treatment by phosphoric acid, which additional treatment will always benefit a diabetic.

As to the admission of alcohol as a remedy, adjuvant or tolerant in the treatment of diabetes, it can do no more than supply to the system a radical or base of assimilation, which has been obtained by its distillation, and which the body of a diabetic has failed to rectify in the carbohydrate taken as food. In other words, the fermented saccharine solution by distillation has hastened the carbohydrates beyond the point of splitting obtainable by the metabolism within the body of the diabetic, and thus it becomes a food product within the system, furnishing the heat and energy necessary to convert into tissue-building material the proteids. This explains the body increase in weight by the admission of alcohol as a remedy in diabetes mellitus, but it does not alter the diseased faulty metabolism. It is known as a physiological fact that a certain amount of alcohol (℥i to ℥ii per day), taken into the system during health, will be appropriated by the animal economy as food, even in addition to a surfeit of the carbohydrates and proteins in the daily food supply of the body. It is said to also allay thirst; this is true of its administration, in that it furnishes food supply, carrying its own oxidizing properties in its inherency, and being sufficiently able to contribute the heat and energy commensurable with its incorporation into body tissue; hence there is no call for $H_2O$ while the alcohol is in a state of assimilation. But does alcohol aid in a cure? My observation and experience in the prescribing and admission of alcohol as a remedy, adjuvant or tolerant, in diabetes mellitus is against it as a beneficial remedy; in fact, it seems to hasten those annoying and serious conditions, pruritus, anthrax, neuralgias, diabetic coma, and the —end. It does not alter the faulty metabolism, or change to normal the deranged functions, which evidences that it is but a—deceiver.

In order to cure our diabetics, the treatment should be given with a view to altering the faulty metabolic condition. The deranged functions of the animal economy must be restored to normal and made to act in harmony with each other. The viscera of the body play no mean part in the metabolism of diabetes, and any remedy or drug which will set in harmonious motion the normal functions of each viscus will by so doing equalize and tranquilize the vaso-motor through each viscus and hence through the cardiac plexus. When this is done all the carbohydrates taken into the system as food will be split up into carbonic acid and water within the animal economy, and diabetes mellitus will cease to—mellitus.

Have we such a drug or remedy or combination of drugs? First, we should consider the etiology, and we are sometimes able to diagnose diabetes as a sequela to some existing or pre-existing disease which may have been acquired or inherited. If syphilis be the predisposing factor, the iodine as a remedy becomes our sheet anchor toward a positive cure; if rheumatic or gouty diatheses, then both iodine and salicylates with colchicum.

In diabetes mellitus there is always a permanent glycosuria which varies in amount of sugar. The danger line will be found above a 2 per cent output. As the percentage of sugar increases, so do the symptoms become more aggravated. There is greater thirst with increased polyuria; the lessened sexual desire may terminate in absolute impotency. The pruritus becomes more annoying, the anthrax more painful by multiple boils and carbuncles, while the tired and sleepy feeling may terminate in diabetic coma.

I have treated a number of cases of diabetes mellitus in my forty years of practice, and have not found it difficult to clear the urine of sugar and cure the patient, since I have better understood the disease, than is prescribed by medical text-books. I first look well into the cause. If syphilitic, I push iodine in some form to a finish; if rheumatic or gouty diatheses, I push iodine, salicylate and colchicum; if obscure, I obtain toward a correction of blood dyscrasia; to altering faulty metabolism; to the re-establishment of the functions of the viscera to normal and in supplying the system with oxygen to aid oxidation, and to this end I prescribe saliodin and alterate with sulphuric acid well diluted with water.

PRESCRIPTION.

℞ Sulphuric acid gtts. x to xx t. i. d. in large glass water immediately after meals. Saliodin grs. xv to xx in water or kouseals twice a day—half way between breakfast and dinner, and half way between dinner and supper. The patient may continue the carbohydrates with his food and yet expect a cure from the above prescriptions.

I have known the sugar in the urine to lessen one-half to twothirds within seventy-two hours from beginning the above treatment, and cease entirely within three to four weeks, proving that the physiological functions are restored to normal by this drug therapy.

MODUS OPERANDI.

Sulphuric acid ($H_2SO_4$) oxidizes the carbohydrates within the animal economy, splitting them up by its reagent qualities into carbonic acid and water, while the saliodin acting upon the stigmata and viscera within the body, the blood and the emunctories restores every function to normal, each ingredient within the compound playing well its part. The salicylate acting as a solvent to the urates and other poisonous compounds. The iodine as an all-efficient eliminant of these poisons from the blood. The acetate acts as a diuretic, flushing the kidneys; the aconite allaying inflammation and controlling fever and thirst; the bryonia acting on the serous and the colchicum on the senovial membranes, while the capsicum and gaultheria aid the salicylate and colchicum. Attention must be given to the bowels. If constipation exists, diabetic coma may supervene.

In the usual treatment of diabetes mellitus when the carbohydrates are withdrawn too long from the food, the proteids split up into carbohydrates, while there are formed from the proteid and fatty foods certain toxic acid substances, such as oxybutyric and diacetic acids, which abstract alkalies, chiefly ammonia, from the tissues. If the proper eliminants are not prescribed, these toxic acid substances produce diabetic coma; therefore, we must always eliminate, especially by way of the alimentary canal, for which give aloin, podophyllin and belladonna at bed time, with an occasional morning flush with Seidlitz powder.

In diabetic coma give soda bicarb. in large doses, pushed to neutralization of the poisonous acid substances within the system. Sig.: By mouth, rectum, or, better, intravenously.

As regards the sulphuric acid administration, I know many physicians will think to find their patient dead after first xx drop dose; but let me assure you there is no danger in double or thrice this amount, providing each dose is sufficiently diluted with water to overcome its escharotic properties before drinking. For thirty years I have prescribed it in malaria and diabetes, and peculiar stomach troubles, and find it the treatment—ne plus ultra. It supplies oxygen to the system, being $H_2SO_4$, and, when $H_2O$ is added, supplies a product to the system which becomes ozonic. I

use it as a test for sugar, and find it both delicate and accurate, and, if it will oxidize the carbohydrates in our laboratory's test tube, why will it not, with even greater efficiency, do likewise within the laboratory of the body's alimentary tube? Besides, there is need of oxygen within the system to aid oxidation; therefore, give $H_2SO_4$ in diabetes mellitus immediately after eating, or, better, half the dose before and the remainder after.

### PROGNOSIS.

This treatment in connection with saliodin and phosphoric acid, paying special attention to the bowels, skin and regimen, will avert those very annoying and serious conditions, and soon restore your diabetic patient to normal health and bring praise and credit to your efficiency and skill.

